# Dysregulation of SNX1-retromer axis in pharmacogenetic models of Parkinson’s disease

**DOI:** 10.1038/s41420-024-02062-8

**Published:** 2024-06-17

**Authors:** Shun Yoshida, Takafumi Hasegawa, Takaaki Nakamura, Kazuki Sato, Naoto Sugeno, Shun Ishiyama, Kiyotoshi Sekiguchi, Muneshige Tobita, Atsushi Takeda, Masashi Aoki

**Affiliations:** 1 Division of Neurology, Department of Neuroscience & Sensory Organs, Tohoku University Graduate School of Medicine, Sendai, Miyagi 980-8574 Japan; 2 Department of Neurology, NHO Yonezawa National Hospital, Yonezawa, Yamagata 992-1202 Japan; 3 Department of Neurology, NHO Sendai-Nishitaga Hospital, Sendai, Miyagi 982-8555 Japan; 4 Department of Neurology, NHO Miyagi National Hospital, Watari, Miyagi 989-2202 Japan; 5 Division of Matrixome Research and Application, Institute for Protein Research, Osaka University, Suita, Osaka 565-0871 Japan

**Keywords:** Parkinson's disease, Mechanisms of disease

## Abstract

Since the identification of vacuolar protein sorting (VPS) 35, as a causative molecule for familial Parkinson’s disease (PD), retromer-mediated endosomal machinery has been a rising factor in the pathogenesis of the disease. The retromer complex cooperates with sorting nexin (SNX) dimer and DNAJC13, another causal molecule in PD, to transport cargoes from endosomes to the trans-Golgi network, and is also involved in mitochondrial dynamics and autophagy. Retromer dysfunction may induce neuronal death leading to PD via several biological cascades, including misfolded, insoluble α-synuclein (aS) accumulation and mitochondrial dysfunction; however, the detailed mechanisms remain poorly understood. In this study, we showed that the stagnation of retromer-mediated retrograde transport consistently occurs in different PD-mimetic conditions, i.e., overexpression of PD-linked mutant DNAJC13, excess aS induction, or toxin-induced mitochondrial dysfunction. Mechanistically, DNAJC13 was found to be involved in clathrin-dependent retromer transport as a functional modulator of SNX1 together with heat shock cognate 70 kDa protein (Hsc70), which was controlled by the binding and dissociation of DNAJC13 and SNX1 in an Hsc70 activity-dependent manner. In addition, excess amount of aS decreased the interaction between SNX1 and VPS35, the core component of retromer. Furthermore, R33, a pharmacological retromer chaperone, reduced insoluble aS and mitigated rotenone-induced neuronal apoptosis. These findings suggest that retrograde transport regulated by SNX1-retromer may be profoundly involved in the pathogenesis of PD and is a potential target for disease-modifying therapy for the disease.

## Introduction

The pathological hallmark of Parkinson’s disease (PD) is the loss of dopaminergic neurons and the appearance of neuronal inclusions mainly composed of α-synuclein (aS) [1]. Mounting evidence has postulated different pathophysiological events as the molecular mechanisms for the development of PD, e.g., mitochondrial dysfunction, oxidative stress, neuroinflammation, and disruption of protein quality control machinery [2]. However, recent genetic approaches in the investigation of the sporadic and familial forms of PD have revealed that molecules involved in endosomal transport play an important role in the pathogenesis thereof [2, 3]. In particular, after the discovery of vacuolar protein sorting 35 (VPS35), a core retromer component, as the causative molecule of PARK17 hereditary PD, the relationship between PD and retromer function has attracted much attention [4, 5]. Furthermore, the overexpression of VPS35 improved the phenotype of transgenic LRRK2 (PARK8) mutant flies and aS transgenic mouse models, indicating that retromer may serve as a common cellular pathway in PD pathogenesis [6, 7].

Retromer is a highly conserved protein complex across species with the vacuolar protein sorting trimer (VPS35, VPS29, and VPS26) as the core components, which cooperates with sorting nexin (SNX) dimer, a protein family containing the phox homology domain that plays an essential role in retrograde transport from endosomes to the *trans*-Golgi network (TGN) [8]. SNX regulates cargo sorting through the generation of curvature and tubular extensions on the endosomal membrane [9, 10]. Other than endosomal trafficking, retromer has multifaceted roles including mitochondrial dynamics and autophagy function [11–14]. The PD-linked mutant VPS35 causes retromer dysfunction which results in neuronal cell death through several mechanisms including insufficient aS degradation and mitochondrial dysfunction [15–17]. Interestingly, growing evidence has suggested that other PD-related proteins are also involved in retromer functioning [18]. For example, aS affects retromer-mediated transport through the inhibition of SNX3 recruitment to the endosomes [19, 20]. In addition, DNAJC13 (also known as receptor-mediated endocytosis 8; RME8), a causal molecule of mutations in the PARK21 familial PD, interacts with SNX1 and VPS35 to regulate retromer functioning [21–23]. Specifically, DNAJC13 regulates cargo transport including mannose-6-phosphate receptor (M6PR) from early endosomes to the TGN through clathrin disassembly, and DNAJC13 as well as SNX1 knockdown impeded the retrograde transport of M6PR in parallel with aberrant accumulation of clathrin in early endosomes [24, 25]. Intriguingly, compared to the wildtype (wt) DNAJC13 (DNAJC13^wt^), the PD-linked mutant DNAJC13 (p.N855S, DNAJC13^N855S^) did not disrupt its association with SNX1 but altered the formation of the SNX1-enriched endosome tubules [26]. Furthermore, the overexpression of VPS35 ameliorated the PD phenotype of several models including aS transgenic mice and mitochondrial toxin (rotenone)-induced toxicity in *Drosophila* [6, 7]. Attempts are also underway to discover disease-modifying drugs that target the retromer in neurodegenerative diseases. For example, retromer stabilization by a pharmacological chaperone improved the neurodegeneration in Alzheimer’s disease (AD) mouse model and human induced pluripotent stem cell (hiPSC)-derived neurons from patients with AD [27, 28]. Similarly, the retromer stabilizer improved locomotor activity and survival rate of motor neurons in amyotrophic lateral sclerosis (ALS) mouse model [29].

Thus, retromer is considered a common pathological mechanism not only in PD but also in other neurodegenerative diseases, making it an attractive target for the development of novel drugs. However, the precise role of retromer function in the molecular pathogenesis of PD remains unclear, and there are many areas that require further investigation. Given these situations, we performed detailed observations on the assembly of the retromer components and impairment of the cargo transport under different PD-mimetic conditions such as, expression of the PD-related DNAJC13 mutant, excess aS, and toxin-induced mitochondrial dysfunction. In addition, we attempted to verify the protective effect of R33, a small molecule retromer stabilizer, against insoluble aS accumulation and rotenone-induced neurotoxicity.

## Results

### PD-linked mutant DNAJC13 causes endosomal enlargement and perturbs retrograde cargo transport from early endosomes to the TGN

**Fig. 1:**
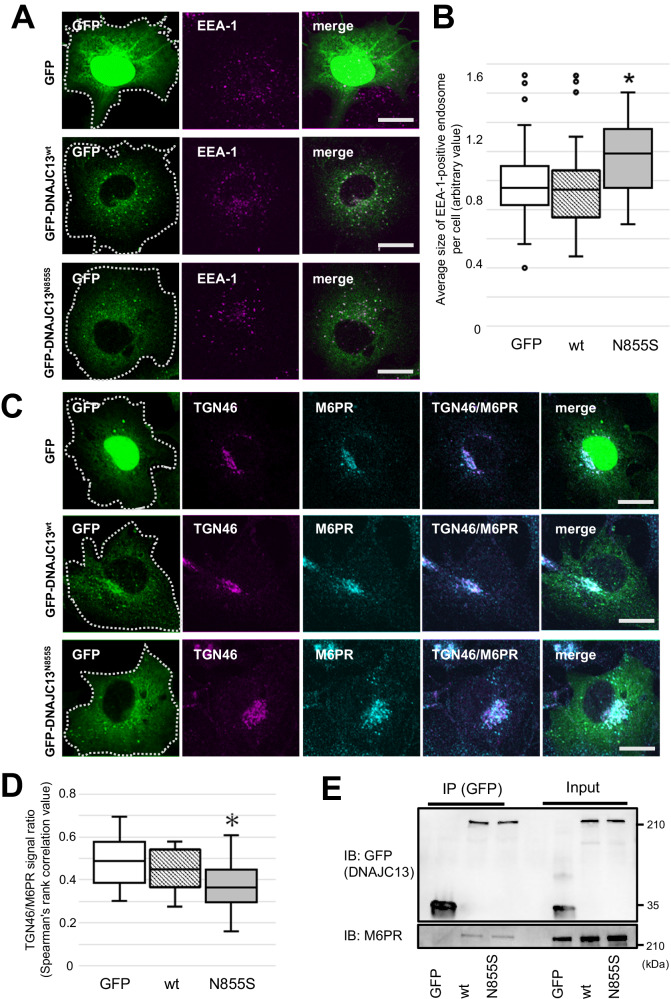
PD-linked mutant DNAJC13 perturbs the retrograde cargo trafficking from early endosomes to the TGN. **A** The COS7 cells overexpressing GFP, GFP-DNAJC13^wt^, or GFP-DNAJC13^N855S^ (green) were immunostained with the early endosomal marker EEA1 (magenta) and images were observed using confocal laser microscopy. Both GFP-DNAJC13^wt^ and GFP-DNAJC13^N855S^ colocalized with the EEA1-positive early endosomes. In cells expressing GFP alone showed artificial localization in the nucleus. Cell contours are indicated by the white dotted lines. Scale bar: 20 μm. **B** The average size of the EEA1-positive endosomes in the cells expressing GFP-DNAJC13^N855S^ was significantly larger than that of GFP or GFP-DNAJC13^wt^. Data were statistically analyzed by Kruskal–Wallis and post hoc Bonferroni tests. *p < 0.05, *n* = 33 (GFP), 32 (GFP-DNAJC13^wt^), and 36 (GFP-DNAJC13^N855S^). **C** The COS7 cells overexpressing GFP, GFP-DNAJC13^wt^, or GFP-DNAJC13^N855S^ (green) were double-immunostained with TGN46 (magenta) and M6PR (cyan) and images were captured using confocal laser microscopy. Cell contours are indicated by the white dotted lines. Scale bar: 20 μm. **D** The colocalization ratio of TGN46 and M6PR was significantly lower in the cells expressing GFP-DNAJC13^N855S^ than in those expressing GFP alone or GFP-DNAJC13^wt^. Data were statistically analyzed by Kruskal–Wallis and post hoc Bonferroni tests. *p < 0.05, *n* = 23 (GFP), 26 (GFP-DNAJC13^wt^), and 29 (GFP-DNAJC13^N855S^). **E** Co-IP analysis of the DNAJC13-M6PR interaction using COS7 cells. Both GFP-DNAJC13^wt^ and GFP-DNAJC13^N855S^, but not GFP alone, bind M6PR to the same extent.

Previously, we showed that the overexpression of the PD-linked DNAJC13^N855S^ mutant protein in COS7 cells perturbed the transport of the epidermal growth factor receptor from early to late endosomes and transferrin to the cell surface [30]. To examine these findings in detail, we tested whether the differences in the morphology of early endosomes could be observed in COS7 cells overexpressing GFP-tagged DNAJC13^wt^ and DNAJC13^N855S^. As previously reported, both DNAJC13^wt^ and DNAJC13^N855S^ localized to the early endosome antigen 1 (EEA1)-positive early endosomes; however, in cells expressing DNAJC13^N855S^, the EEA1-positive endosomes were enlarged compared to the cells expressing GFP alone or DNAJC13^wt^ (Fig. 1A, B). Several lines of evidence have demonstrated that DNAJC13 regulates the cargo transport in multiple directions, starting from early to late endosomes, cell surface, and the TGN. However, there is no clear evidence that DNAJC13^N855S^ impairs the retromer-mediated retrograde transport from the early endosomes to TGN [31–33]. To confirm this, we used M6PR as a reference molecule for retromer transport and observed its co-localization with a TGN marker, TGN46, in the DNAJC13 expressing cells. Interestingly, the co-localization of M6PR and TGN46 was significantly reduced in cells expressing DNAJC13^N855S^ compared to those expressing GFP alone or DNAJC13^wt^ (Fig. 1C, D). The co-IP showed no significant difference in the interaction with M6PR between the DNAJC13^wt^ and DNAJC13^N855S^ expressing cells (Fig. 1E), indicating that the altered subcellular distribution of M6PR was not attributable to the PD-linked pathogenic DNAJC13 mutation.

### Chlorpromazine inhibits endosomal tubulation, increases interaction between DNAJC13 and SNX1, and inhibits retrograde transport by retromer

**Fig. 2:**
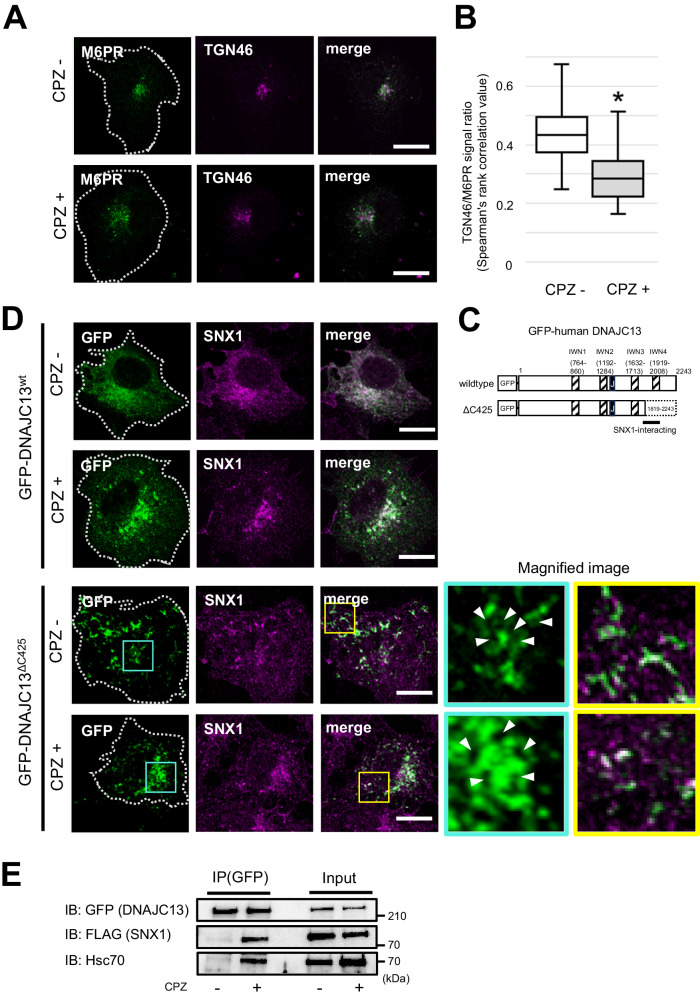
Chlorpromazine inhibits retromer-mediated transport and endosomal tube formation, increases the interaction between DNAJC13 and SNX1. **A** Image of CPZ-treated COS7 cells that were co-immunostained with M6PR (green) and TGN46 (magenta) and observed using a confocal laser microscope; in the CPZ-treated cells, M6PR appears scattered throughout the cytoplasm. The white dotted lines indicate outline of the cells. Scale bar: 20 μm. **B** Co-localization ratio of M6PR and TGN46 in the CPZ-treated cells was significantly lesser than that of untreated cells. Data were statistically analyzed by Mann–Whitney *U* test. *p < 0.05, *n* = 24 (untreated group) and 20 (CPZ-treated group). **C** Domain architecture of GFP-tagged human DNAJC13 used in this study. It contains four domains of repetitive amino acid sequences (IWN = isoleucine (I), tryptophan (W) and asparagine (N), indicated as shaded squares), with the DNAJ domain (black box) it interacts with HSC70/HSP70. The PD-linked N855S mutation is located within the first IWN domain. The black line indicates the presumed SNX1 binding site [21, 36]. **D** Confocal images of COS7 cells expressing GFP-DNAJC13^wt^ or GFP-DNAJC13^ΔC425^ (green) co-immunostained with SNX1 (magenta). GFP-DNAJC13^wt^ was well colocalized with SNX1, while the coexistence of GFP-DNAJC13^ΔC425^ and SNX1 was somewhat reduced, and accompanied by vacuolar formation (white arrowheads in the cyan-framed inset) and SNX1-negative tubular structures (magnified image framed in yellow). After CPZ exposure, more GFP-DNAJC13^ΔC425^ coexisted with SNX1 and SNX1-negative tubular structures tended to shorten. The white dotted lines indicate outline of the cells. Scale bar: 20 μm. **E** Molecular interaction of GFP-DNAJC13^wt^ with FLAG-SNX1 and Hsc70 in the presence or absence of CPZ was confirmed by co-IP. CPZ treatment markedly increased the binding of SNX1 and Hsc70 to GFP-DNAJC13^wt^.

Previous studies using HeLa cells have shown that clathrin induces budding on the endosomal membrane in cooperation with DNAJC13 for cargo sorting by retromer [24, 34]. To examine the effect of clathrin on the retromer-DNAJC13-mediated cargo transport, a pharmacological inhibition model was adopted. To this end, we used COS7 cells exposed to chlorpromazine (CPZ, 30 μM for 30 min), which prevents the assembly and disassembly of the clathrin lattice on the cell surface and endosomal membrane [35], and observed the changes in the co-localization of M6PR and TGN46. In the cells exposed to CPZ, M6PR tended to be scattered throughout the cytoplasm, and the co-localization rate with TGN46 was significantly lower than that of the untreated cells (Fig. 2A, B). Structurally, DNAJC13 is composed of an N-terminal PI(3)P lipid-binding domain, DNAJ domain, which coordinates Hsp70/Hsc70 binding, and a C-terminal SNX1-binding domain [36]. In addition, four IWN repeats of the central portion where two each are located on either side of the DNAJ domain. The DNAJ domain recruits Hsc70 to activate the ATPase function, thereby regulating its proper folding/refolding, oligomerization, and translocation across the membrane. A recent study using budding yeast and *Caenorhabditis elegans* (*C. elegans*) has suggested that the Hsc70-unbound RME-8 (a human DNAJC13 homolog) forms oligomers by physically interacting with its own DNAJ domain and the C-terminal IWN3 and IWN4 domains, thereby masking the DNAJ domain and inhibiting its interaction with Hsc70 [36]. It has been suggested that SNX1 physically interacts with the C-terminus of RME-8 to de-oligomerize RME-8 and promotes binding of the DNAJ domain to Hsc70, thus regulating RME-8 activation and distribution in the endosomal microdomain [36]. In a study using HeLa cells, the Bin/Amphiphysin/Rvs domain of SNX1 was implicated in endosomal membrane budding and tubulation [9]. Based on these findings, we investigated how clathrin affects the endosomal membrane remodeling as well as the physical binding of DNAJC13 and SNX1. First, we exposed cells expressing GFP-tagged DNAJC13^wt^ or C-terminal truncated mutant ΔC425 DNAJC13 (DNAJC13^ΔC425^) lacking the IWN4 domain (Fig. 2C) to CPZ and examined the SNX1 subcellular localization. The subcellular localization of DNAJC13^wt^ corresponded with that of SNX1, while DNAJC13^ΔC425^ colocalized only partially with SNX1, demonstrating abnormal vacuolar formation and SNX1-negative tubular structures (Fig. 2D). Somewhat surprisingly, CPZ treatment markedly increased the co-localization of DNAJC13^ΔC425^ and SNX1, with the concomitant shortening of SNX1-negative tubular structures (Fig. 2D). Similar to CPZ exposure, silencing of clathrin heavy chain also shortened the tubular structures, suggesting that clathrin triggers tubular deformation of endosomal membrane (Fig. S1). Consistent with these results, the co-IP analysis using cells expressing both GFP-tagged DNAJC13^wt^ and FLAG-tagged SNX1 showed that the binding between DNAJC13 and SNX1 was significantly enhanced in the presence of CPZ. It is also interesting to note that CPZ enhanced the total and DNAJC13-bound Hsc70. (Fig. 2E).

### Retromer-mediated cargo transport is regulated by Hsc70 activity-dependent binding and dissociation of DNAJC13 from SNX1

**Fig. 3:**
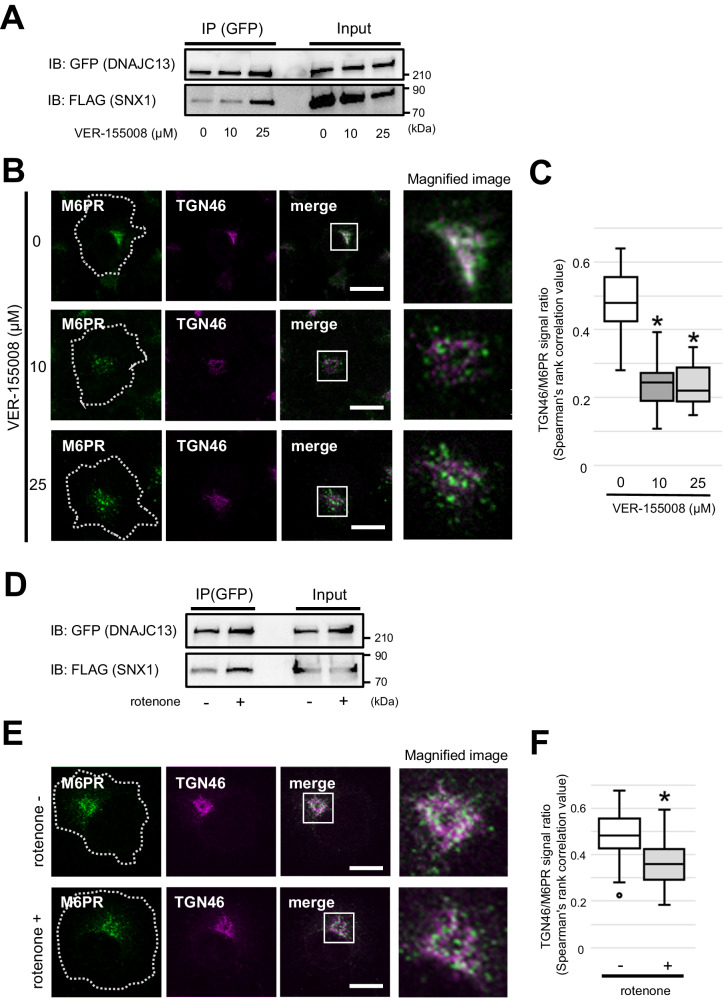
Retromer-mediated cargo transport is regulated by Hsc70 activity-dependent binding and dissociation of DNAJC13 from SNX1. **A** Co-IP analysis of GFP-DNAJC13^wt^ and FLAG-SNX1 in COS7 cells exposed to different concentrations of the Hsc70 inhibitor VER-155008 for 24 h. Treatment with VER-155008 markedly increased the binding of DNAJC13 and SNX1 in a dose-dependent manner. **B** Confocal images of the COS7 cells exposed to VER-155008 co-immunostained with M6PR (green) and TGN46 (magenta). The VER-155008 treated cells show more dispersed punctate distribution of M6PR compared to the untreated cells. The white dotted lines indicate outline of the cells. The inset shows a magnified image of the white square. Scale bar: 20 μm. **C** Co-localization ratio of M6PR and TGN46 in VER-155008-treated cells is significantly less than that of the untreated cells. Data were statistically analyzed using the Kruskal–Wallis and post hoc Bonferroni tests. *p < 0.05, *n* = 31 (0 μM), 30 (10 μM), and 28 (25 μM). **D** Co-IP analysis of GFP-DNAJC13^wt^ and FLAG-SNX1 in COS7 cells in the presence or absence of rotenone treatment at a final concentration of 0.1 μM for 3 days. Binding of SNX1 to DNAJC13 was enhanced with rotenone treatment. **E** Confocal images of the rotenone-treated cells co-immunostained with M6PR (green) and TGN46 (magenta). The white dotted lines indicate the cell outlines. The inset shows a magnified image of the white square. Scale bar: 20 μm. **F** Co-localization ratio of M6PR and TGN46 was lower in the rotenone-treated cells than in the untreated cells. Data were statistically analyzed by Mann–Whitney *U* test. *p < 0.05, *n* = 36 (untreated group) and 36 (rotenone-treated group).

Given that CPZ increased the total amount of Hsc70 and the interaction between SNX1 and DNAJC13, DNAJC13 may act as a functional modulator of SNX1 along with Hsc70. To confirm this, we used VER-155008, a pharmacological Hsc70 inhibitor, and observed the changes in the binding status of DNAJC13 and SNX1 through co-IP in COS7 cells. VER-155008 substantially increased the interaction between SNX1 and DNAJC13 in a dose-dependent manner (Fig. 3A). Next, we observed changes in the subcellular localization of M6PR and TGN46 in cells exposed to VER-155008. After the treatment with VER-155008, the M6PR-positive puncta were dispersed near the cell periphery, and the colocalization rate with the TGN46-positive structures was reduced (Fig. 3B, C). As a ubiquitous and highly conserved cytosolic chaperone, Hsp70/Hsc70 assists numerous proteins to adopt a native conformational state or recover function after misfolding in an ATP-dependent manner, and the primary source of the ATP used for this is from the mitochondria [37]. Thus, we investigated the physical interaction between DNAJC13 and SNX1 and its effect on the subcellular localization of M6PR using cells treated with the mitochondrial complex I inhibitor, rotenone, a dopaminergic neurotoxin widely used to generate a pharmacological PD model [38]. Intriguingly, similar to VER-155008, rotenone reinforced the interaction between DNAJC13 and SNX1 compared to the mock treatment and slightly, but significantly, reduced the co-localization rate at M6PR and TGN46 (Fig. 3D–F). These results suggest that retromer-mediated cargo sorting may be regulated through the Hsc70 activity-dependent binding and dissociation of DNAJC13 from SNX1.

### Excess aS reduces the interaction between SNX1 and VPS35 and perturbs the retrograde cargo transport

**Fig. 4:**
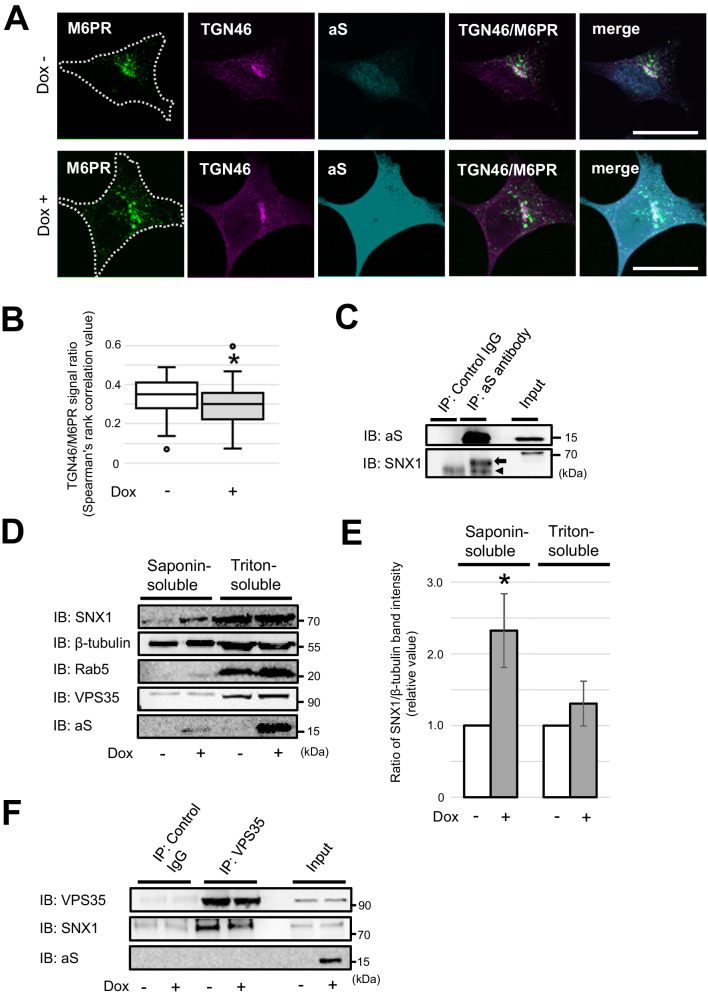
Excess aS decreases the interaction between SNX1 and VPS35 and inhibits the retrograde transport. **A** Confocal microscopic images of Dox-inducible aS-expressing SH-SY5Y cells triple-immunostained for M6PR (green), TGN46 (magenta), and aS (cyan). Upon aS induction, M6PR showed a more dispersed distribution near the cell periphery compared to the uninduced cells. The white dotted line indicates the cell periphery. Scale bar: 20 μm. **B** The colocalization rate of M6PR and TGN46 was slightly, but significantly, reduced by aS induction. Data were statistically analyzed using the Mann–Whitney *U* test. *p < 0.05, *n* = 51 (non-induced) and 51 (induced). **C** Co-IP analysis of binding between aS and SNX1 in the cytoplasmic fraction of the aS-induced cells. The arrow and arrowhead indicate the bands of SNX1 and IgG heavy chain, respectively. **D** Results of the WB analysis of the aS-induced and non-induced cells solubilized with saponin and Triton X- 100. The early endosomal marker Rab5 was detected in the Triton- but not the saponin-soluble fraction, indicating that the former mainly contains cytoplasmic proteins and the latter membrane-associated components. Upon aS induction, SNX1 in the saponin-soluble cytosolic fraction increased, while SNX1 in the triton-soluble membrane fraction remained unchanged. β-tubulin is an indicator of equal loading. **E** The ratio of SNX1/β-tubulin band intensity. Values of Dox-induced cells are expressed relative to the values of Dox-uninduced cells which are presented as 1.0, and the calculated values are expressed as the mean ± standard error. Data were statistically analyzed using the Student’s *t* test. *p < 0.05, *n* = 4. **F** Interaction between VPS35 and SNX1 before and after aS induction and verified by co-IP. The amount of SNX1 bound to VPS35 was slightly decreased after the aS induction.

Although several reports making use of different cellular and animal models have shown that retromer dysfunction is responsible for the intracellular accumulation of aS, only a few reported data have shown that the excessive amount of aS itself results in retromer dysfunction [15, 39]. The latter possibility is worth considering since aS gene is known to cause familial PD not only through missense mutations but also by gene duplication/triplication, indicating that even wt aS can lead to neurodegeneration when present in excess [40, 41]. Experiments using budding yeast have shown that the overexpression of aS disrupts retromer sorting via the inhibition of SNX3 recruitment to the endosomes [19, 20]. Based on these findings, we examined whether the presence of excess wt aS could affect the retrograde cargo transport using SH-SY5Y cells in which wt aS is inducible through the addition of Dox. Interestingly, in the aS-induced cells, M6PR decreased in co-localization with TGN46 (Fig. 4A, B). A previous study showed that aS interacts physically and genetically with SNX1 but not with VPS35 [42]. Thus, we investigated the interaction between aS and SNX1 via the co-IP of the cytoplasmic fraction extracted from SH-SY5Y cells permeabilized with saponin, a mild detergent [43]. Consistent with previous reports, the interaction between aS and SNX1 was reproduced (Fig. 4C). Following this, quantitative analysis by Western blot (WB) was performed to evaluate the changes in the SNX1 protein levels in the cytoplasmic and membrane-associated fractions with and without the induction of aS expression. The results showed that the amount of SNX1 in the saponin-soluble fraction was significantly increased by the increased levels of aS expression (Fig. 4D, E). The excess amount of aS may hamper the translocation of SNX1 from the cytosolic to the membrane fraction containing the endosomes. Similarly, there was a slight decrease in the amount of SNX1 interacting with VPS35 when aS expression was induced (Fig. 4F), suggesting that the excessive amount of aS might affect the SNX1 recruitment to the retromer complex.

### Retromer stabilization reduces the amount of insoluble aS and protects against rotenone-induced cytotoxicity in neuronal cells

**Fig. 5:**
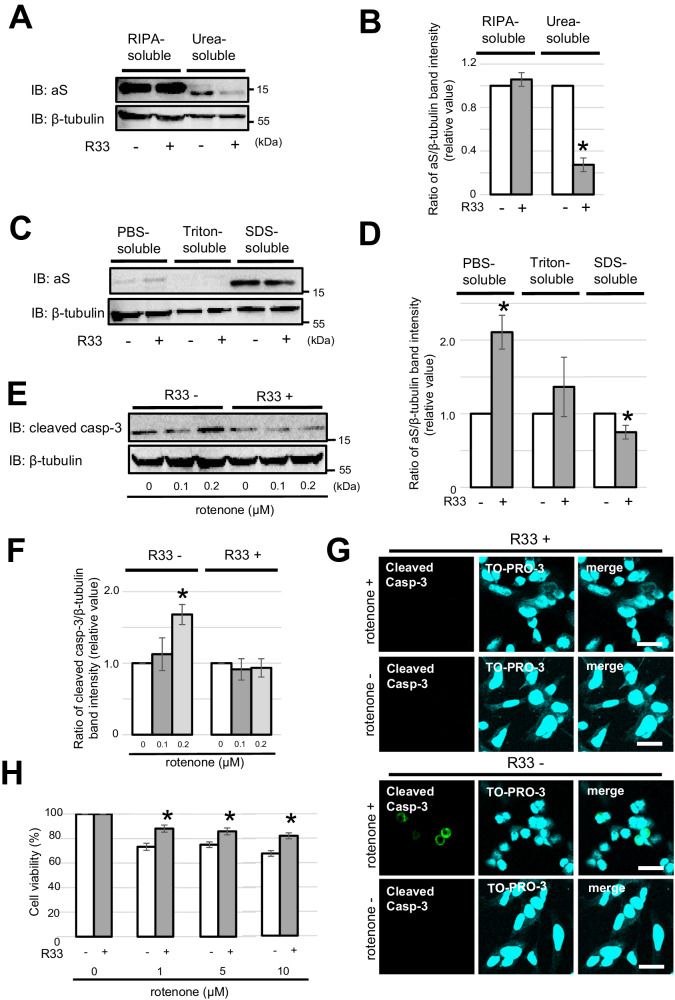
Retromer stabilization reduced the amount of insoluble aS and alleviates rotenone-induced cytotoxicity. **A** SH-SY5Y cells preincubated for 4 days with a concentration of 5 μM R33, a retromer stabilizer, were sequentially solubilization with RIPA and urea buffer, and analyzed by WB. R33 treatment did not change the amount of RIPA-soluble aS but decreased the RIPA-insoluble, urea-soluble aS level. β-tubulin was used as the loading control. **B** Comparison of aS/β-tubulin signal ratio in the RIPA- and urea-soluble fractions. Values of the R33-treated cells are expressed relative to the values of the R33-untreated cells which are preincubated as 1.0, and the calculated values are expressed as the mean ± standard error. Data are statistically analyzed using the Student’s *t* test. *p < 0.05, *n* = 4. **C** SH-SY5Y cells before and after R33 treatment were sequentially solubilized with PBS-, Triton X-100-, and SDS-buffer, followed by WB analysis. Treatment with R33 increased the PBS-soluble aS levels and slightly, but significantly, decreased the amount of Triton-insoluble, SDS-soluble aS. **D** Comparison of the aS/β-tubulin signal ratio in PBS-, Triton X-100-, and SDS-soluble fractions. Values of the R33-treated cells are expressed relative to the values of R33-untreated cells which are presented as 1.0, and the calculated values are expressed as the mean ± standard error. Data were statistically analyzed using the Student’s *t* test. *p < 0.05, *n* = 4. **E** SH-SY5Y cells with and without R33 pretreatment were exposed to rotenone for 24 h, and cleaved caspase-3 expression levels were evaluated through WB analysis. The level of cleaved caspase-3 remained lower in the R33-pretreated cells than in the untreated cells. **F** Signal ratio of cleaved caspase-3/β-tubulin in the WB analysis after rotenone exposure. Rotenone exposure (0.2 μM) significantly increased the expression levels of cleaved caspase-3 compared to baseline of the R33 untreated group. However, in the R33 preincubated group, the cleaved caspase-3 expression level remained unchanged. Values of rotenone-treated cells are expressed relative to the values for the rotenone-untreated cells which are presented as 1.0, and the calculated values are expressed as the mean ± standard error. Data were statistically analyzed by one-way ANOVA followed by the Bonferroni post hoc test. *p < 0.05, *n* = 4. **G** SH-SY5Y cells with and without R33 pretreatment were exposed to rotenone and immunostained with cleaved caspase-3 antibody. Cleaved caspase-3 signal (green) induced by rotenone was hardly detected in the R33-treated cells. Nuclei were counterstained with TO-PRO-3 (cyan). Scale bar: 20 μm. **H** SH-SY5Y cells with and without R33 pretreatment were exposed to rotenone for 24 h and cell survival was confirmed using an MTT assay. R33-pretreatment significantly improved cell survival. Data were statistically analyzed using two-way ANOVA test and the post hoc Bonferroni test. Quantitative data are expressed as the mean ± standard error. *p < 0.05, *n* = 6.

Consistent evidence of retromer dysfunction in the different PD models, e.g., mutant DNAJC13 expression, mitochondrial toxicity, and excessive aS induction, implies that the retromer may be a target for disease-modifying therapy in PD. To test this hypothesis, SH-SY5Y cells were treated with R33, a retromer-stabilizing chemical chaperone, and changes in the expression of insoluble aS were evaluated by WB [44]. As shown in Fig. 5A, B, the amount of aS in the urea-soluble-RIPA buffer-insoluble fraction was increased in the R33-treated cells, while the levels of aS in RIPA-soluble fraction with and without R33 treatment did not differ significantly. To further investigate this change in the aS solubility after R33 treatment, cells were sequentially solubilized with different buffers containing phosphate-buffered saline (PBS), Triton X-100, and SDS, and subjected to immunoblot analysis. Interestingly, the addition of R33 resulted in a slight but significant decrease in the amount of aS in the Triton-insoluble-SDS-soluble fraction, while the aS level in the PBS-soluble fraction showed a moderate increase (Fig. 5C, D). Furthermore, we investigated the cytoprotective effect of R33 against the rotenone-induced apoptosis in SH-SY5Y cells [45]. The WB analysis of SH-SY5Y cells exposed to rotenone showed that the level of cleaved caspase-3 was significantly increased in the cells without R33 treatment but not in the cells that were preincubated with R33 (Fig. 5E, F). Consistent with this result, immunostaining for cleaved caspase-3 also revealed its presence in cells without R33 treatment, while the R33-treated cells did not demonstrate a positive signal (Fig. 5G). Similarly, R33 showed a significant protective effect against rotenone-induced cytotoxicity via the cell viability evaluated using an MTT assay (Fig. 5H).

## Discussion

After the discovery of *VPS35* as the causative gene of the PARK17 familial form of PD, the involvement of retromers in the pathogenesis of PD has received particular attention [4, 5, 18]. While many studies in this research area have used VPS35 as the starting point, research on the other retromer-related molecules such as DNAJC13 still remains limited. Both familial PD due to VPS35 and DNAJC13 mutations present with typical dopa-responsive parkinsonism due to the loss of dopaminergic neurons in the midbrain; however, aS-positive Lewy bodies only appear in the latter [4, 5, 22, 46]. In addition, the overexpression of PD-linked D620N mutant VPS35 increases the levels of abnormally phosphorylated aS in mice brain; however, endogenous aS is dispensable for nigrostriatal dopaminergic neurodegeneration [47]. Our previous study using a transgenic aS fly model demonstrated that the co-expression of DNAJC13^N855S^ promoted the locomotor dysfunction and dopaminergic neurodegeneration with the accumulation of detergent-insoluble aS, which suggests that the pathogenic DNAJC13 mutant contributes to the buildup of aS pathology [30]. Given that DNAJC13 binds to the early endosome surface via its N-terminal region to regulate the multi-directional cargo transport, the abnormally enlarged early endosomes resulting from DNAJC13^N855S^ expression in this study can be interpreted as a result of the accumulation of the retained cargo in early endosomes [25, 31, 33]. Indeed, the addition of transferrin to COS7 cells overexpressing DNAJC13^N855S^ increased the size of early endosomes compared to the DNAJC13^wt^ expressing cells [22].

In addition to previous data, which showed that DNAJC13^N855S^ perturbed the cargo transport from early endosomes to the cell surface or late endosomes, the present study provided corroborating evidence that the retromer-mediated retrograde transport from early endosomes to the TGN was impaired as well. Mechanistically, it has been reported that DNAJC13^N855S^ altered the SNX1-positive endosomal tubule formation in mouse primary cortical neurons. Furthermore, in a transgenic worm model, SNX1 was uncoated from the RME8 on endosomes in an Hsc70-dependent manner [26, 36]. In agreement with this report, we confirmed that the retromer-mediated retrograde transport of M6PR was regulated through the Hsc70 activity-dependent binding and dissociation of DNAJC13 from SNX1. DNAJC13^N855S^ may disrupt the Hsc70-mediated SNX1 dynamics, but the amount of SNX1 interacting with DNAJC13 was not significantly different between DNAJC13^wt^ and DNAJC13^N855S^, suggesting a different mechanism other than the impaired molecular interaction is involved [26]. Intriguingly, several lines of evidence have postulated that DNAJC13 functions as a positive autophagy modulator. Specifically, in *C. elegans* and cultured cell models, the knockdown of RME8/DNAJC13 reduced the autophagic activity by impairing the ATG9A trafficking and/or autolysosome reformation processes. In line with this, DNAJC13^N855S^-expressing cells failed to show the enhanced autophagic flux that was observed by the overexpression of DNAJC13^wt^, which suggests that the DNAJC13^N855S^ mutant may act in a dominant-negative fashion [48, 49].

Another important factor in the endosomal trafficking regulated by DNAJC13 is clathrin. Clathrin serves as a major component of the protein coat of certain post-Golgi and endocytic vesicles [50]. The silencing of RME8, a DNAJC13 homolog in *C. elegans*, resulted in the aberrant accumulation of clathrin on the endosomes, leading to the inhibition of its transport from early endosomes [21, 25]. Importantly, evidence suggests that several familial PD-related molecules other than DNAJC13 are also involved in the clathrin-mediated endocytosis. For example, auxilin (also known as DNAJC6), whose mutation causes early-onset PD (PARK19), functions as a cofactor for clathrin uncoating by Hsc70 [51, 52]. In addition, LRRK2 mutants with elevated kinase activity reduced the clathrin-dependent endocytosis via the phosphorylation of endophilin A1 and auxilin [53–57]. Moreover, aS dimers and excess aS accumulation impaired the clathrin-dependent endosomal recycling at the synapses [58, 59]. Considering the present results that rotenone inhibits the dissociation of SNX1 from DNAJC13 as well as Hsc70 inhibitors, it is possible that the clathrin-mediated functional coupling between DNAJC13 and SNX1 may be involved in the pathophysiological cascades of PD.

The first indication suggesting that mitochondrial dysfunction in the pathogenesis of PD was a report of parkinsonism due to 1-methyl-4-phenyl-1,2,3,6-tetrahydropyridine (MPTP), a mitochondrial toxin, poisoning [60]. Subsequently, brain autopsies of patients with PD were also found to have substantially reduced complex I activity, which led to the widespread recognition of mitochondrial dysfunction in PD [61]. Rotenone, a complex I inhibitor, induces the selective toxicity to dopamine neurons and the chronic systemic administration of low dose rotenone to rodents reproduced the selective dopaminergic neurodegeneration and the formation of Lewy body-like inclusions, making this compound a versatile tool to generate experimental PD models [62–64]. Increasing evidence has shown that retromer dysfunction leads to mitochondrial failure [14, 16]; however, there are few reports demonstrating how mitochondrial dysfunction affects the retromer. In this context, the present results showing that rotenone inhibits cargo transport by retromers are noteworthy and merit further study. As mentioned above, we demonstrated that Hsc70 inhibition, like rotenone, suppressed the SNX1 dynamics through DNAJC13 and caused retromer dysfunction. Interestingly, in the brains of patients with PD, the Hsc70 protein level was reduced and correlated with the increased levels of aS, while the accumulation of aS decreased the Hsc70 levels at the synapses [65]. These findings suggest a potentially functional link between SNX1 dynamics and aS via Hsc70 chaperone activity in PD pathology. Our findings that aS inhibits the retromer-mediated transport of SNX1 via the inhibition of its recruitment to endosomes provides evidence for the importance of SNX1 in the molecular basis of PD.

Given the present results that the chemical chaperone R33, which stabilizes retromer, reduced the amount of insoluble aS and decreased the rotenone-induced cytotoxicity, retromer may be a promising disease-modifying therapeutic target for PD. This is supported by previous studies in which the deletion of VPS35 in yeast impaired the transport of VPS10 (a human sortilin homolog) from the endosomes to the TGN, resulting in the accumulation of aggregated proteins because of transport failure from the TGN to the degradation pathway [7]. It is also interesting in the context of our recent finding that aS fibrils were bound to sortilin on the cell surface and transported via the endolysosomal pathway [66]. The unexpected result in the present study, that the solubility of aS was altered by R33, is worth further discussion. We previously observed a shift in the intracellular aS from the PBS soluble to insoluble fraction with the overexpression of the DNAJC13 PD mutant, indicating that the alteration of retromer function may affect the aS solubility [30]. However, the overexpression of VPS35 in *Drosophila*, which lacks endogenous aS, successfully reduced the rotenone toxicity, suggesting that there may be independent mechanisms involved in the aS expression changes due to retromer function and rotenone-induced cytotoxicity [6]. Importantly, the retromer has also been implicated in the pathological mechanisms of neurodegenerative diseases other than PD: e.g., VPS35 and VPS26 were reduced at both the mRNA and protein levels in the entorhinal cortex of patients with AD [67]. In addition, the stabilization of retromers with chemical chaperones in AD mouse models and hiPSCs reduced the Aβ deposition and phosphorylated tau [27, 28]. Furthermore, the expression level of VPS35 was reduced in the autopsied spinal cord and familial ALS-associated mutant SOD1 transgenic mice [29, 68], while retromer stabilizers improved the phenotype in SOD1-transgenic mice [29]. It will be of great interest to further analyze the role of the retromer transport in neurodegeneration, and to gain further insight into the mechanisms by which the retromer stabilization confers neuroprotection in a variety of neurodegenerative diseases.

## Materials and methods

### Cell culture, plasmid preparation, and transfection

COS7 cells (RCB0539; RIKEN BRC, Tsukuba, Japan) and SH-SY5Y dopaminergic cells (CRL-2266; ATCC, Manassas, VA, USA) in which human wt aS is inducible by doxycycline (Dox) were maintained in high-glucose Dulbecco’s modified Eagle’s medium (FUJIFILM Wako Chemicals, Osaka, Japan) supplemented with 10% fetal bovine serum (HyClone Laboratories, Logan, UT, USA) at 37 °C in humidified 5% CO_2_/air [69]. The mammalian expression vectors pAAV-CAG/GFP- wt DNAJC13 and pAAV-CAG/GFP-N855S mutant DNAJC13 (NM015268) were kindly gifted from Dr. Matthew J. Farrer (University of British Columbia, Vancouver, Canada). pEGFP-C1/ΔC425 DNAJC13 was prepared as reported previously [33]. Human wt SNX1 cDNA (NM003099) was purchased from Kazusa DNA Research Institute (Chiba, Japan) and subcloned into p3xFLAG-CMV-10 vector. Plasmids were transfected using the NEPA Gene^TM^ square-wave electroporator (NEPA Gene, Ichikawa, Japan).

### Immunocytochemical analysis

Cells were fixed with 4% paraformaldehyde (PFA) in PBS for 20 min, permeabilized with 0.1% Triton X-100 in PBS for 5 min, and blocked 3% bovine serum albumin in PBS for 30 min. Primary antibodies used were as follows: anti-EEA1 (3288; CST, Danvers, MA, USA), anti-TGN46 (AHP500; Bio-Rad, Hercules, CA, USA), anti-SNX1 (ab134126; Abcam, Cambridge, MA, USA), anti-M6PR (ab2733; Abcam), anti-aS (MJFR-1, ab138501; Abcam), and anti-cleaved caspase-3 (9661; CST). The primary antibody reaction was followed by incubation with the following secondary antibodies: anti-mouse IgG Alexa Fluor 488, anti-rabbit IgG Alexa Fluor 488, anti-rabbit IgG Alexa Fluor 555, anti-sheep IgG Alexa Fluor 555, and anti- rabbit IgG Alexa Fluor 647 conjugates (all purchased from Thermo Fisher Scientific, Waltham, MA, USA). Nuclei were counterstained with TO-PRO-3 (Thermo Fisher Scientific/Molecular Probes). Fluorescent images were captured with an FV300 confocal laser scanning microscope (Olympus, Tokyo, Japan). Quantitative colocalization analyses of multicolor confocal immunofluorescence images were performed using image analysis software Fiji (https://fiji.sc).

### Co-immunoprecipitation

Cells were washed twice with PBS and lysed in lysis buffer containing 20 mM HEPES-KOH (pH 7.5), 200 mM sorbitol, 2 mM EDTA, 50 mM potassium acetate, and 0.1% Triton X-100. Lysates were incubated overnight on a carousel at 4 °C with anti-GFP antibody conjugated with magnetic beads (PM035-8; MBL, Tokyo, Japan), anti-aS (Abcam), anti-VPS35 (GTX108058; GeneTex, Irvine, CA, USA) or control IgG (MBL) with Pierce protein A/G magnetic beads (Thermo Fisher Scientific). After being washed 4 times with lysis buffer, the protein complex were eluted with 2× Laemmli buffer at room temperature for 1 h and subjected to Western blot analysis. For co-immunoprecipitation (co-IP) using cytosol fraction, cells were lysed with buffer A [150 mM KCl, 2 mM MgCl2, 20 mM HEPES-KOH (pH 7.5), 10% glycerol, 1 mM dithiothreitol, 1 mM EDTA] containing 0.02% saponin [43].

### Western immunoblot analysis

Lysates prepared from cultured cells were electrophoresed on sodium dodecyl sulfate-polyacrylamide gel electrophoresis (SDS-PAGE) gels and transferred onto polyvinylidene difluoride (PVDF) membranes. After treatment with a Blocking 1 Reagent for Can Get Signal^TM^ (TOYOBO, Osaka, Japan), the membrane was incubated with primary antibody and subsequently reacted with second antibody. The antibodies used in this study were as follows: anti-GFP (598; MBL), anti-M6PR (ab2733; Abcam), anti-FLAG/M2 (F1804; Merck Millipore/SIGMA, Billerica, MA, USA), anti-Hsc70 (SPA-815; Stressgen, Ann Arbor, MI, USA), anti-VPS35 [GTX108058; GeneTex (rabbit) and WH0055737M2; Merck Millipore/SIGMA (mouse)], anti-SNX1 (ab134126; Abcam), anti-β-tubulin (66240-1-Ig; Proteintech, Rosemont, IL, USA), anti-Rab5 (2141; CST), anti-aS (MJFR-1, ab138501; Abcam), anti-cleaved caspase-3 (9661; CST). To improve the detection of aS, the transferred PVDF membranes were soaked in 0.4% PFA/PBS for 20 min prior to the blocking step [70]. The bands were visualized with Luminata Forte^TM^ HRP Substrate (Merck Millipore), and images were captured using an Omega Lum G^TM^ imager system (Aplegen, San Francisco, CA, USA). Quantification of band intensity was performed using the Fiji.

### Isolation of cytosolic and membrane fractions in cultured cells

To separate cytosolic and membrane proteins from cultured cells, we adopted a subcellular fractionation protocol with slight modifications [43]. In brief, cells were lysed with buffer A containing 0.02% (w/v) saponin and incubated for 20 min at 4 °C to allow the release of the cytosolic proteins. The saponin-soluble cytosolic fraction was then centrifuged at 17,000 × *g* for 20 min. The pellet was lysed with buffer A containing 1% Triton X-100 and incubated for 20 min at 4 °C. The lysates were centrifuged at 17,000 × *g* for 20 min to obtain a Triton-soluble membrane fraction. Rab5 was used as a marker for membrane fraction.

### Sequential solubilization of α-synuclein

To detect insoluble aS, cells were lysed with radioimmunoprecipitation assay (RIPA) buffer [1% NP-40, 0.5% deoxycholate, 0.1% SDS, 1 mM EDTA, 10 mM sodium pyrophosphate, 50 mM sodium fluoride, 1 mM sodium orthovanadate, 150 mM sodium chloride, and 50 mM Tris-HCl (pH 8.0)] for 20 min at 4 °C. After centrifugation at 17,000 × *g* for 20 min to obtain a RIPA-soluble fraction, the pellet was dissolved in 8 M urea/5% SDS followed by sonication to obtain RIPA-insoluble fraction. Alternatively, stepwise solubilization under milder condition was performed as follows: cells were first resuspended in PBS and sonicated. After centrifugation at 17,000 × *g* for 20 min to obtain a PBS-soluble fraction, the pellet was dissolved in lysis buffer [150 mM NaCl, 50 mM Tris-HCl (pH 8.0), 1 mM EDTA, 1% Triton X-100] for 20 min at 4 °C. After additional centrifugation at 17,000 × *g* for 20 min to obtain a Triton-soluble fraction, the pellet was dissolved in lysis buffer containing 2% SDS followed by sonication and centrifugated at 17,000 × *g* for 20 min. Each fraction obtained above was subjected to WB to quantify the amount of aS.

### MTT cell viability assay

Cells at approximately 80% confluency were cultured on 96-well plate in 100 μl of medium. After adding 3-(4,5-dimethylthiazol-2-yl)-2,5-diphenyltetrazolium bromide (MTT; Merck Millipore/SIGMA) at a final concentration of 0.45 mg/ml, cells were incubated for 3 h and the medium was carefully removed. Then, 100 μl/well dimethyl sulfoxide was added to dissolve formazan crystals by shaking, and the absorbance at 570 nm was measured using a spectrophotometer (Multiskan™ GO Microplate Spectrophotometer, Thermo Fisher Scientific).

### Statistical analysis

Statistical analyses were performed using R software version 4.2.2 (https://www.R-project.org/).

### Supplementary information

Supplemental Figure S1

uncropped original western blots

## Data Availability

All data are available in the main text and Supplementary Information.
