# Supplementary material for: Dysregulation of SNX1-retromer axis in pharmacogenetic models of Parkinson’s disease
Source: Cell Death Discov. 2024 Jun 17;10:290. doi: 10.1038/s41420-024-02062-8 (PMC11183211; doi:10.1038/s41420-024-02062-8)
Supplement: Supplementary file 1 — Supplemental Figure S1 [file 41420_2024_2062_MOESM1_ESM.pptx]

## Slide 1
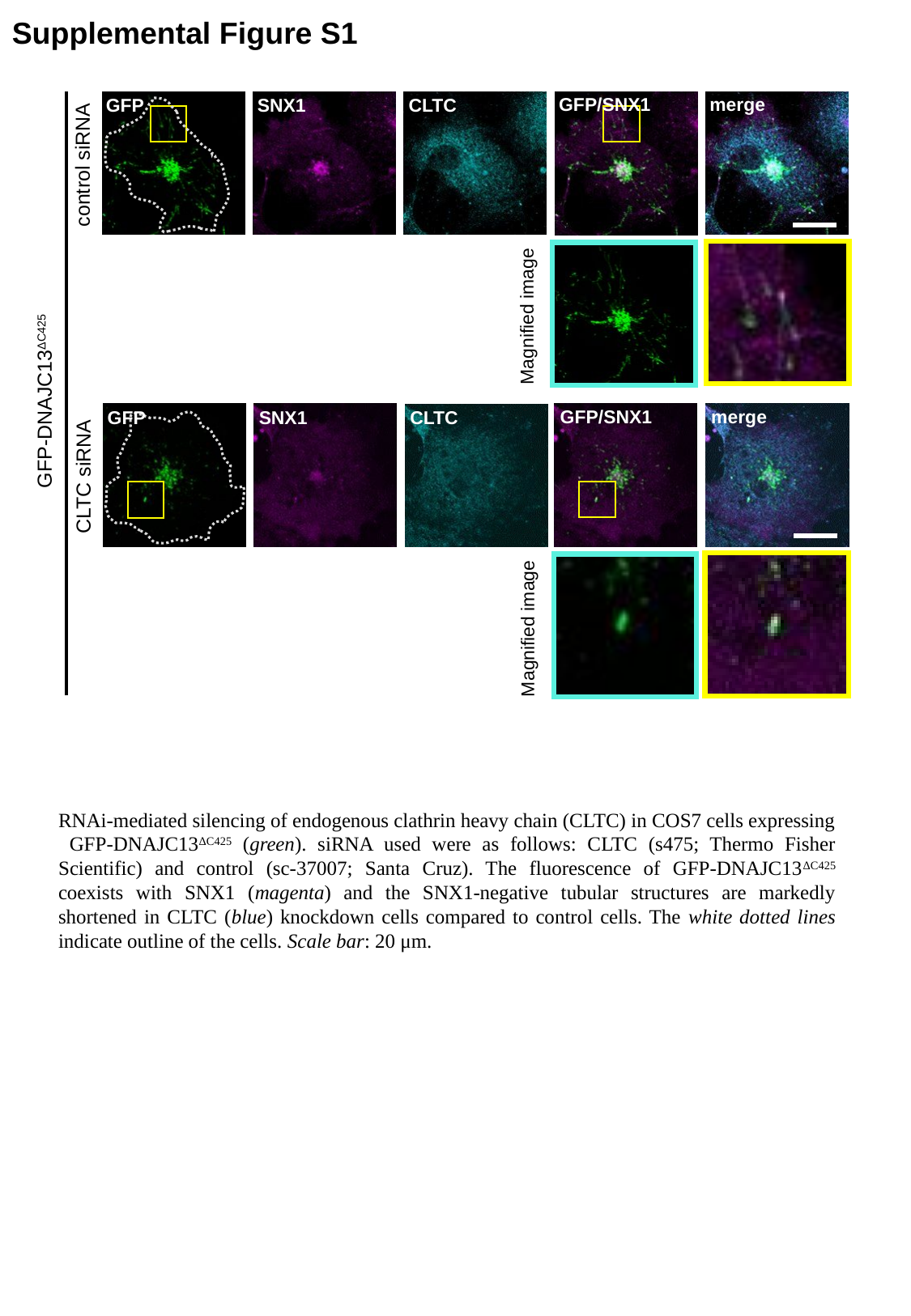

Supplemental Figure S1
GFP/SNX1
merge
GFP
SNX1
CLTC
control siRNA
Magnified image
GFP-DNAJC13ΔC425
GFP/SNX1
merge
GFP
SNX1
CLTC
CLTC siRNA
Magnified image
RNAi-mediated silencing of endogenous clathrin heavy chain (CLTC) in COS7 cells expressing GFP-DNAJC13ΔC425 (green). siRNA used were as follows: CLTC (s475; Thermo Fisher Scientific) and control (sc-37007; Santa Cruz). The fluorescence of GFP-DNAJC13ΔC425 coexists with SNX1 (magenta) and the SNX1-negative tubular structures are markedly shortened in CLTC (blue) knockdown cells compared to control cells. The white dotted lines indicate outline of the cells. Scale bar: 20 μm.
