# Supplementary material for: Dysregulation of SNX1-retromer axis in pharmacogenetic models of Parkinson’s disease
Source: Cell Death Discov. 2024 Jun 17;10:290. doi: 10.1038/s41420-024-02062-8 (PMC11183211; doi:10.1038/s41420-024-02062-8)
Supplement: Supplementary file 2 — uncropped original western blots [file 41420_2024_2062_MOESM2_ESM.pptx]

## Slide 1
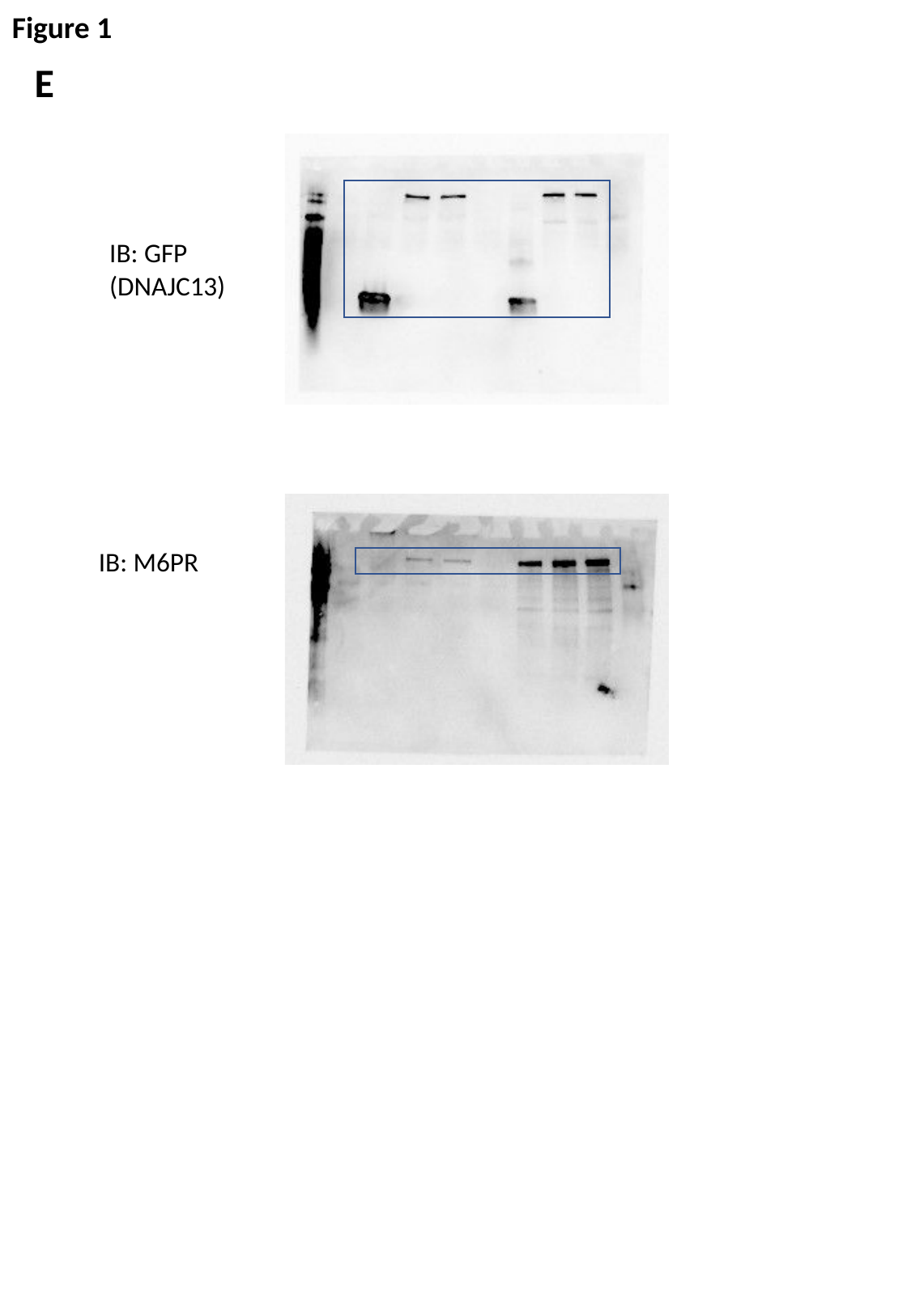

Figure 1
E
IB: GFP
(DNAJC13)
IB: M6PR

## Slide 2
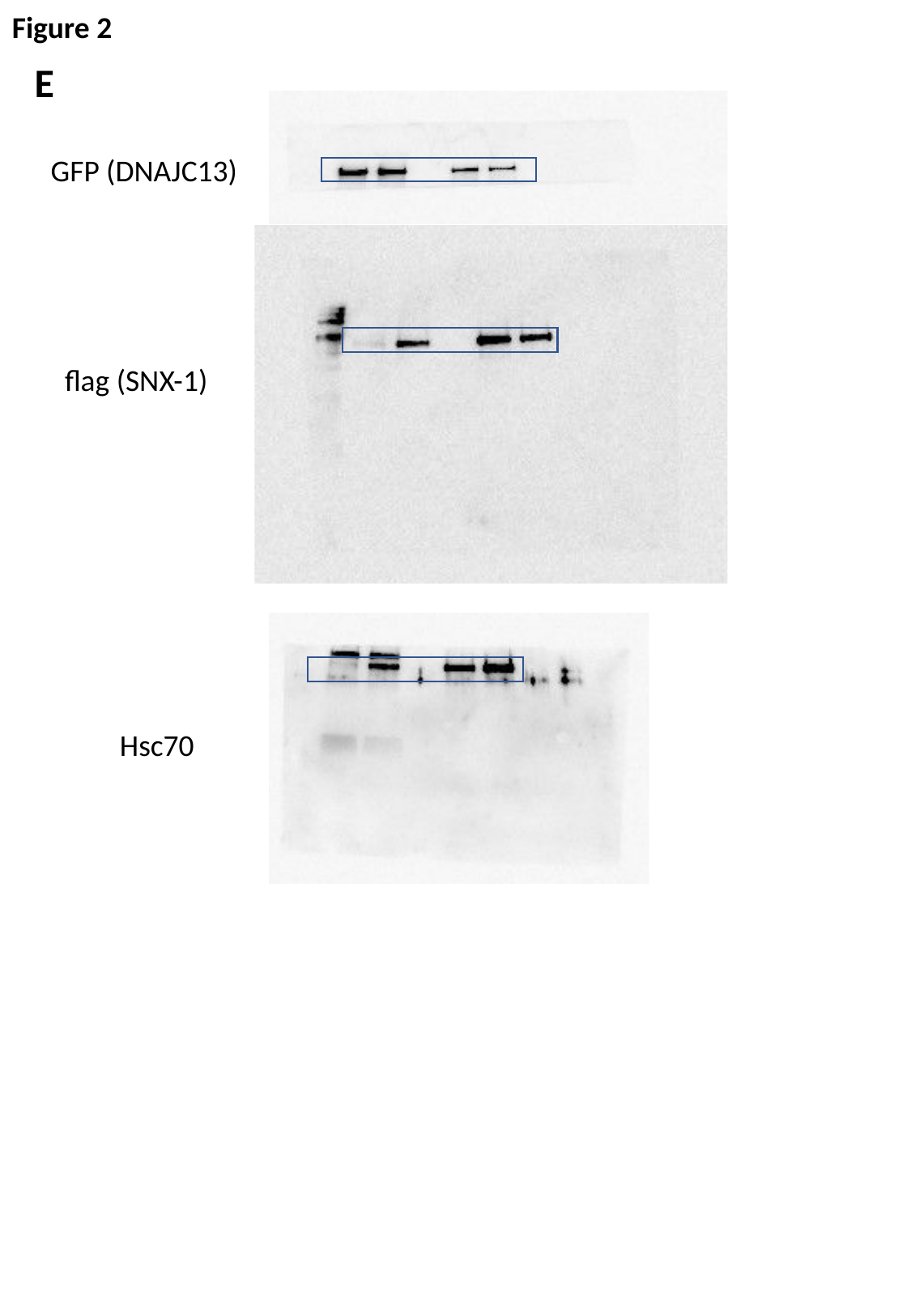

Figure 2
E
GFP (DNAJC13)
flag (SNX-1)
Hsc70

## Slide 3
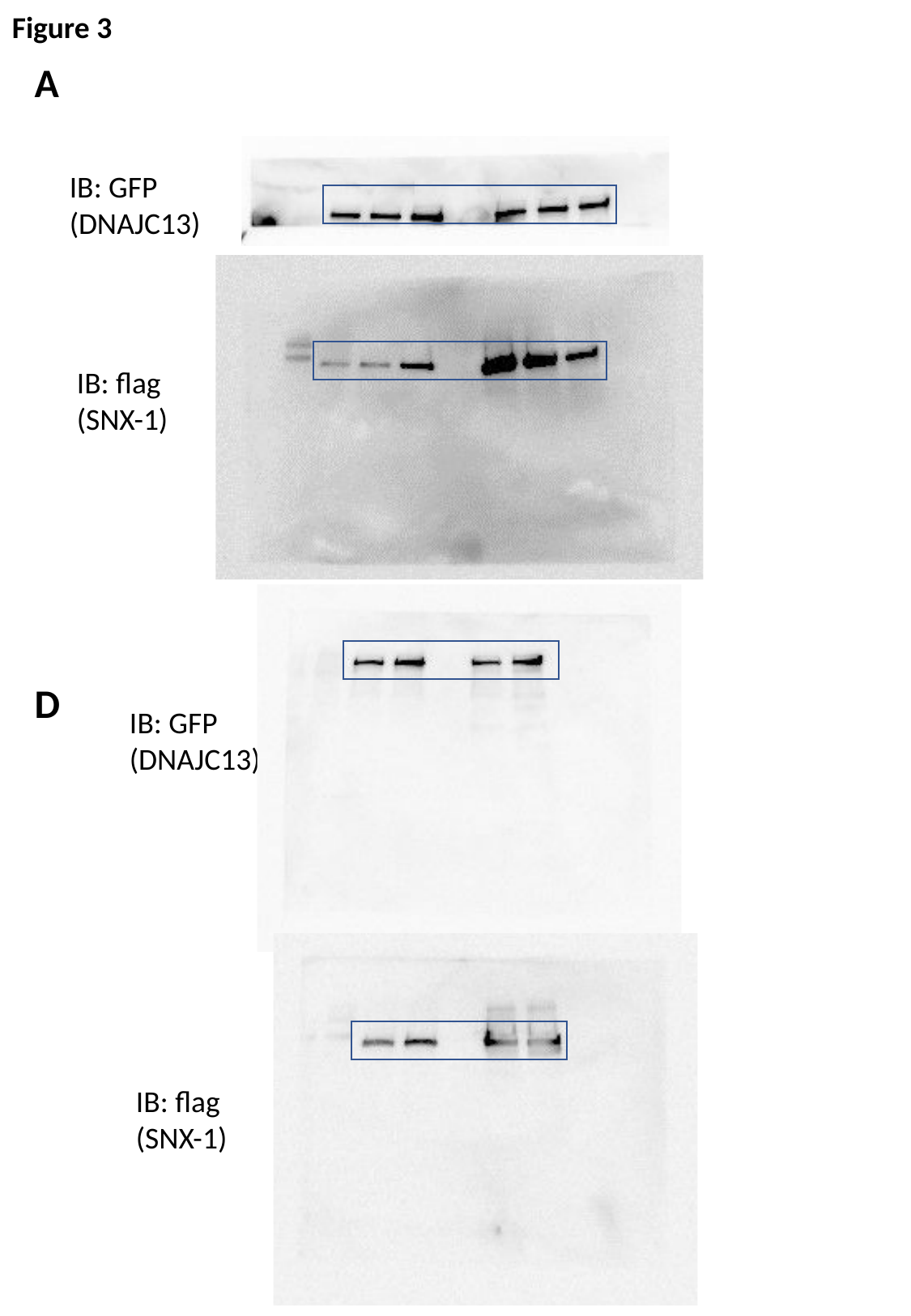

Figure 3
A
IB: GFP
(DNAJC13)
IB: flag
(SNX-1)
D
IB: GFP
(DNAJC13)
IB: flag
(SNX-1)

## Slide 4
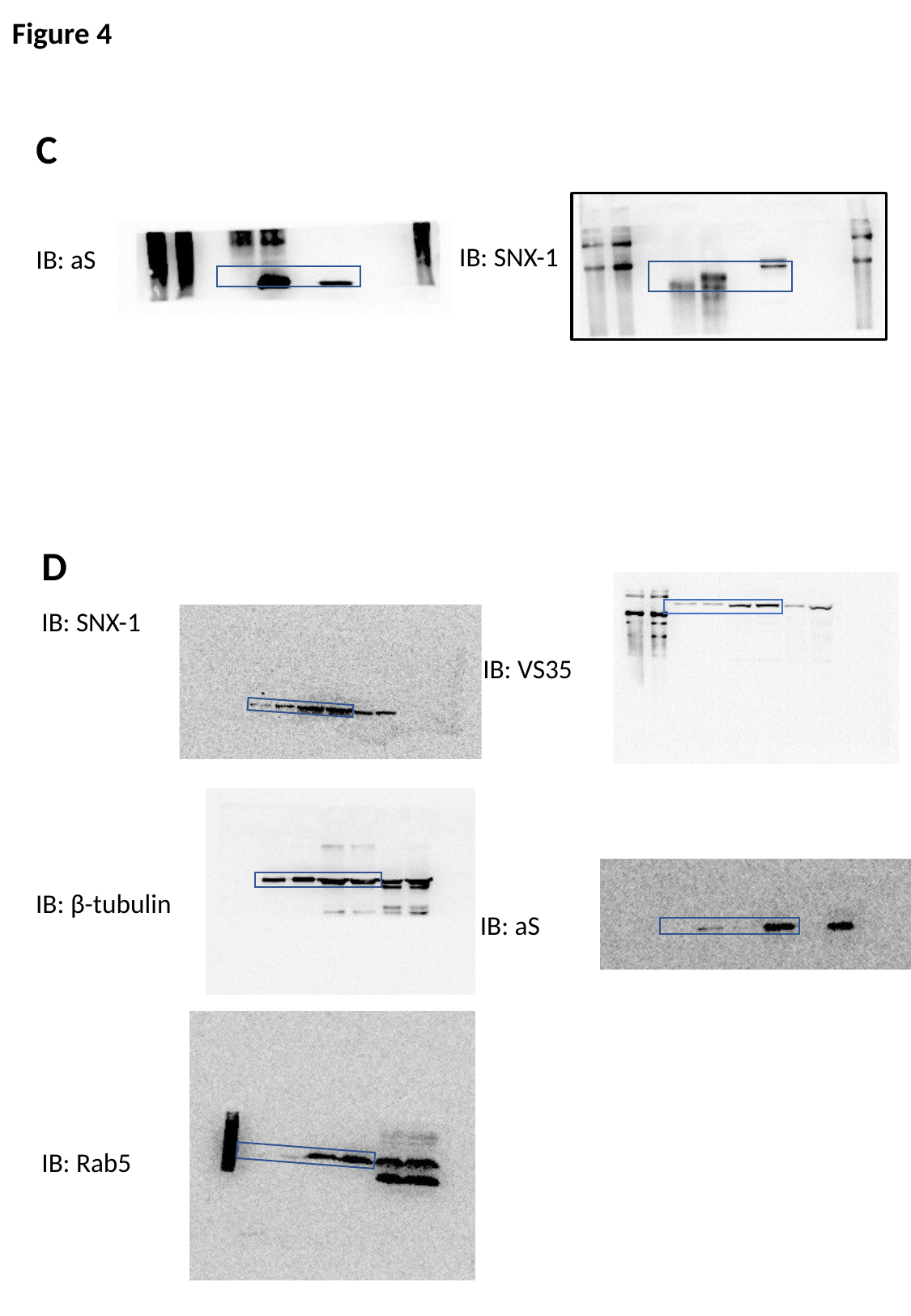

Figure 4
C
IB: SNX-1
IB: aS
D
IB: SNX-1
IB: VS35
IB: β-tubulin
IB: aS
IB: Rab5

## Slide 5
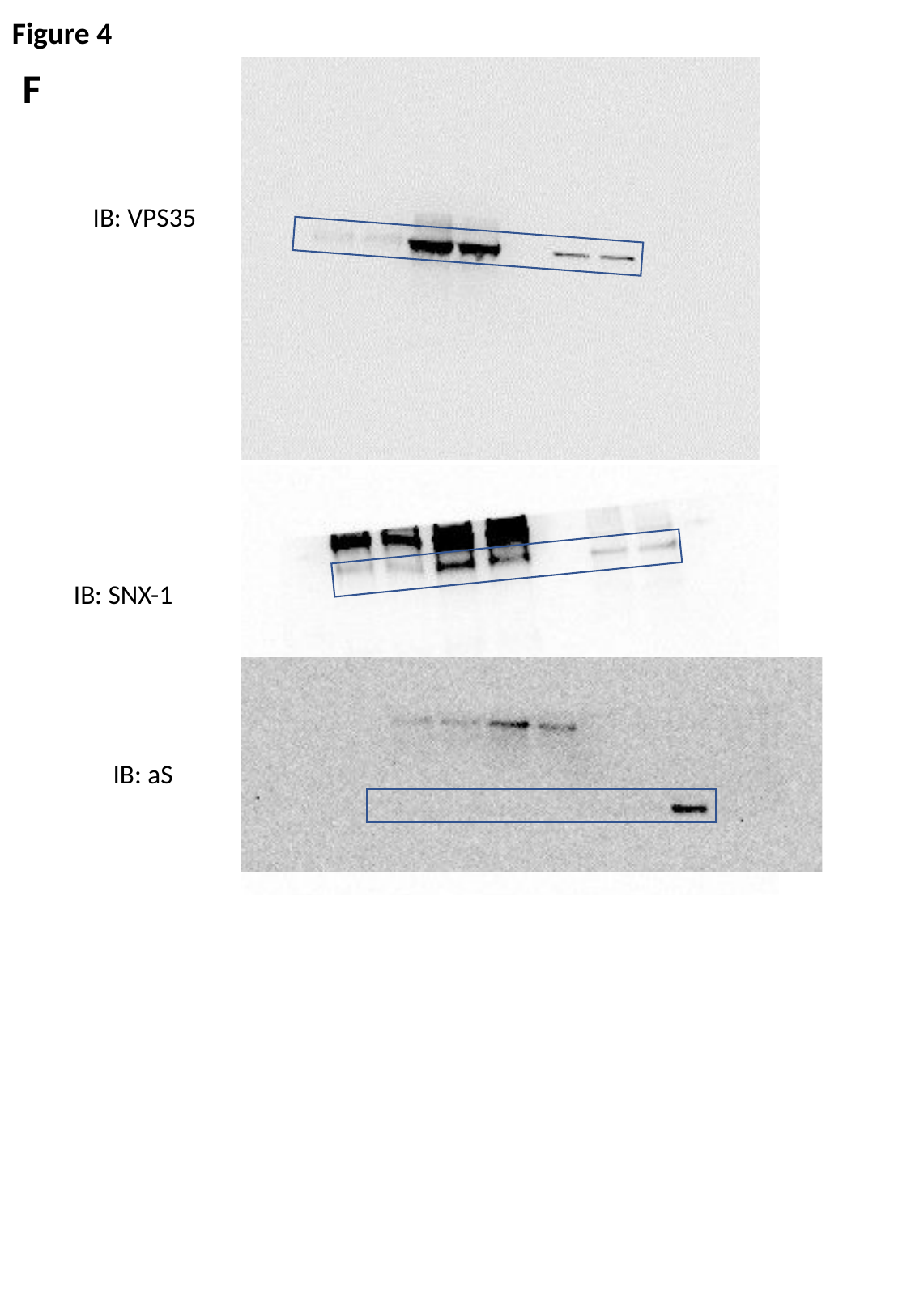

Figure 4
F
IB: VPS35
IB: SNX-1
IB: aS

## Slide 6
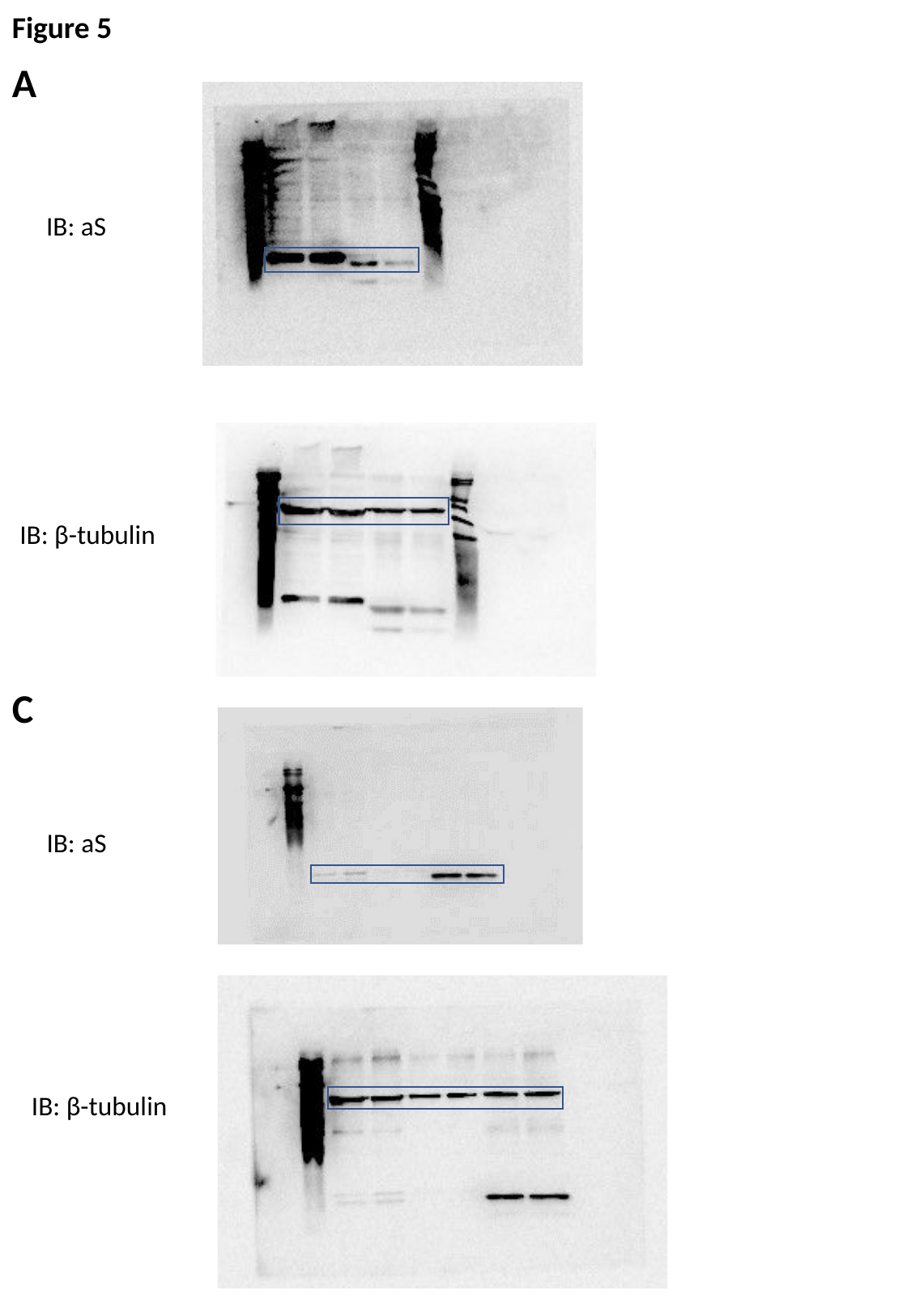

Figure 5
A
IB: aS
IB: β-tubulin
C
IB: aS
IB: β-tubulin

## Slide 7
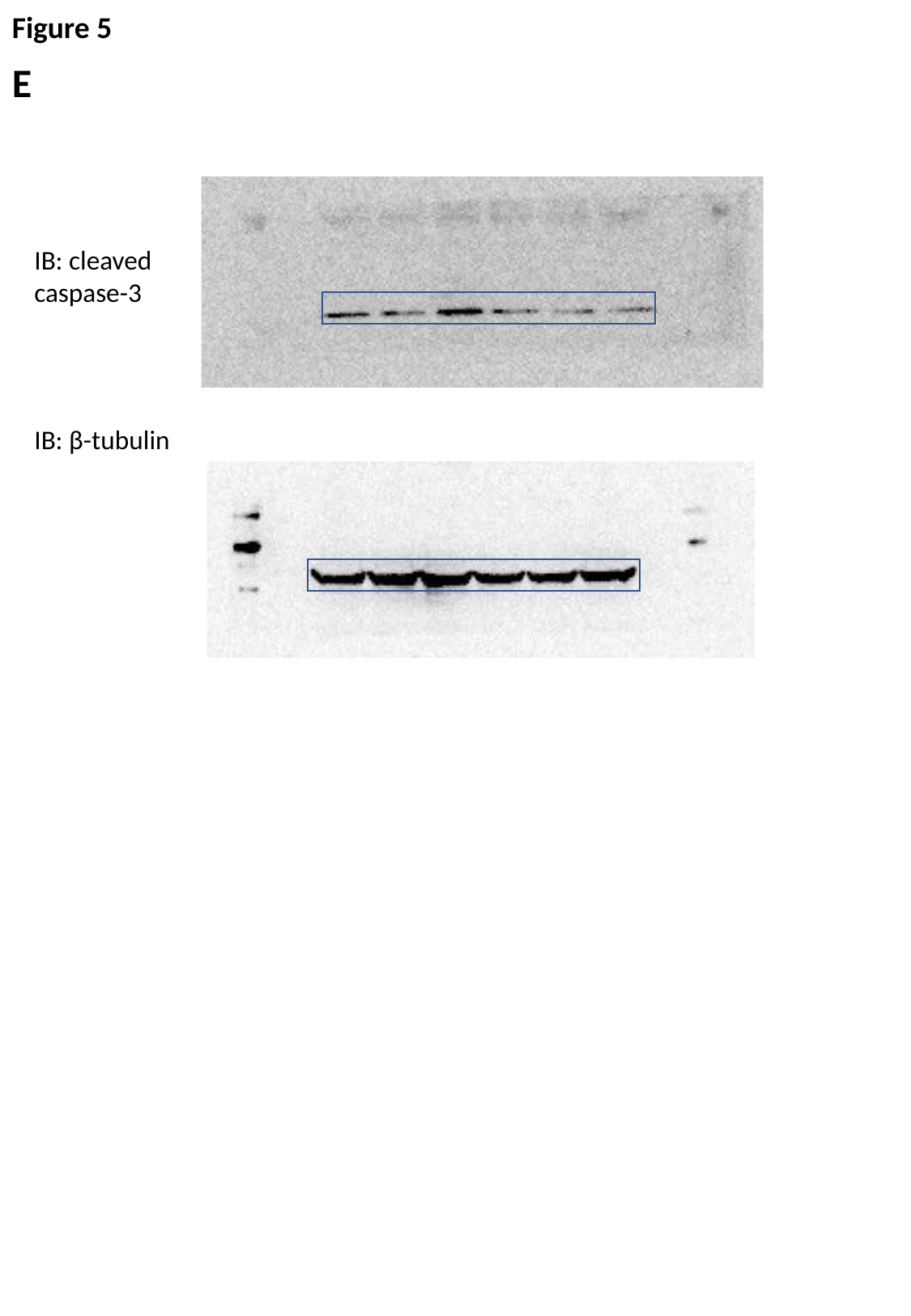

Figure 5
E
IB: cleaved caspase-3
IB: β-tubulin
